# Cytology in Mucinous Breast Carcinoma: Diagnostic Insights and a Rare Bilateral Case

**DOI:** 10.7759/cureus.88670

**Published:** 2025-07-24

**Authors:** Ashish Kawthalkar, Supreeta Nayak, Kirti Jaiswal

**Affiliations:** 1 Pathology, Government Medical College and Hospital, Nagpur, Nagpur, IND

**Keywords:** cytohistologic correlation, cytological criteria, fine-needle aspiration cytology (fnac), metachronous contralateral breast cancer, mucinous breast carcinoma

## Abstract

Introduction

Cytological evaluation of breast lumps is often conducted as part of the triple test for breast carcinomas and provides a confirmation of the clinical and radiological diagnosis. Mucinous carcinomas of the breast, while a relatively rare entity, are important to recognize due to their favorable prognosis. The present research has been designed to study and highlight the specific cytological features of mucinous carcinoma of the breast as well as present a rare case of cytologically diagnosed bilateral breast mucinous carcinoma.

Materials and methods

A two-year retrospective analysis of cytologically diagnosed and histopathologically confirmed cases of mucinous carcinoma of the breast was conducted. Relevant clinical and radiological details were noted and included in the study. All slides were assessed for specific features that assist in the final diagnosis of mucinous carcinoma based on cytology alone.

Results

A total of seven cases of mucinous carcinoma of the breast in a span of two years were identified on cytology. Fine-needle aspiration cytology (FNAC) yielded mucoid material in all cases. Abundant extracellular mucin was observed in all cases. In all cases, there was abundant cellularity. Most of the epithelial cells were seen to be arranged predominantly in clusters. In all cases, cells were uniform and had a moderate amount of cytoplasm and a round nucleus showing mild atypia. Branching, delicate capillary fragments (chicken-wire vascularity) were noted in six (85%) cases. Histopathological correlation was available for all cases, confirming mucinous carcinoma. There was a single case of metachronous bilateral breast mucinous carcinomas, with the right and left breast lesions being palpable five and three years back, respectively, by the patient.

Discussion

The mean size, age group, and cytological features were consistent with the findings of previous studies. Typically, pure mucinous subtypes show abundant extracellular mucin with floating islands and isolated tumor cells within on histopathology. Mixed mucinous carcinomas often show more solid areas with an invasive ductal carcinoma component. In the present study, there was a single case of bilateral mucinous carcinoma of the breast, making it only the third case to be reported so far. To the best of our knowledge, it is the first case of mucinous carcinoma presenting in bilateral breasts to be reported on FNAC.

Conclusion

Strict adherence to the above diagnostic cytologic criteria, which includes abundant pools of mucin, tight clusters of epithelial cells exhibiting mild atypia, and branching capillaries, is the key to a confident diagnosis of mucinous carcinoma of the breast on cytology. Though extremely rare, bilateral mucinous carcinoma of the breast does occur and can be reliably diagnosed on cytology.

## Introduction

Breast carcinomas are the most common malignancies in women, with a disproportionately higher prevalence in Asian women [1]. Cytological evaluation of breast lumps is often one of the components of the famed “triple test” for breast carcinomas, requiring clinical, radiological, and pathological testing of suspicious lumps for satisfactory management of patients [2]. While there are many clinical and radiological features that point towards a diagnosis favoring malignancy, the importance of confirmation and subtyping of the suspected malignancy through pathological means cannot be overstated.

Mucinous carcinoma of the breast is a relatively rare entity, accounting for around 2% of all breast carcinomas. These tumors are usually transcriptionally distinct from usual breast carcinomas and hence have been categorized separately in the WHO classification systems since 2003. There is also a clear association between mucinous carcinomas and lower rates of recurrence. These individuals usually have an excellent long-term prognosis [3].

Awareness of cytological features that assist in early identification of this rare entity is of paramount importance, considering the major prognostic implications. For preliminary diagnosis, fine-needle aspiration cytology (FNAC) is useful, but interpretation should warrant higher caution.

The present research has been designed to study and highlight the specific cytological features of mucinous carcinoma of the breast, as well as present a rare case of cytologically diagnosed bilateral breast mucinous carcinoma.

## Materials and methods

A two-year retrospective analysis of cytologically diagnosed and histopathologically confirmed cases of mucinous carcinoma of the breast was conducted. All FNAs were performed without radiological guidance with the use of a 23-gauge hypodermic needle and a 10 ml syringe. In tumors larger than 2 cm, multiple separate sites were sampled. Cytological slides were stained with hematoxylin & eosin (H&E), Papanicolaou, and May-Grunwald Giemsa (MGG) stain. Relevant clinical and radiological details were noted and included in the study. All slides were assessed for specific features that assist in the final diagnosis of mucinous carcinoma based on cytology alone.

## Results

A total of seven cases of mucinous carcinoma of the breast in a span of two years were identified on cytology. This study includes seven female patients diagnosed with mucinous carcinoma of the breast, aged between 49 and 83 years (mean age: 65.7 years). Two of the patients had two lesions each, totaling nine lesions. The most commonly affected site was the upper outer quadrant, with four lesions involving the left breast and five lesions in the right. The presenting symptom in all cases was a gradually enlarging breast lump. The patients noticed the lump themselves on self-examination. However, the lumps differed from the usual lumps of duct carcinomas by being either firm or firm to hard rather than hard, with most of them also being nonmobile (Table 1).

**Table 1:** Clinical features *UOQ: upper outer quadrant; **LOQ: lower outer quadrant; ***UIQ: upper inner quadrant; ^#^L: left breast; ^##^R: right breast The numerical identifiers (e.g., 1, 2, 3, etc.) used in the table are arbitrary and were created solely for the purpose of referencing specific cases within this article. These identifiers do not correspond to any patient-identifying information.

Case number	Age (years)	Site	Size (cm)	Duration	Clinical finding
Case 1	68	UOQ* – L^#^	4x3.5	2 months	Firm to hard and mobile
Case 2	49	2 lesions - UOQ – L	4.5x4 and 3x2	Both, since 1 year	Larger: firm, nonmobile; Smaller (noted on ultrasound): firm to hard, nontender mobile
Case 3	57	UOQ – R^##^	4x4	5 years	Firm to hard, nonmobile
Case 4	83	UOQ – R	3x3	1.5 years	Firm, nonmobile
Case 5	68	LOQ** – R	3x2	2 months	Firm to hard, nonmobile
Case 6	75	1 – UIQ***– R 2 – UOQ – L	R - 7x7 L - 4x3	R - 5 years. L – 3 years	Both: firm, mobile,
Case 7	60	UOQ – R	4x3	1 year	Firm, nonmobile

Radiological ultrasound and mammography imaging (available in two patients) demonstrated suspicious features (Breast Imaging Reporting and Data System-Category 5 (BIRADS 5)) with hypoechoic heterogeneous lesions, and microlobulation was noted in one case. Unfortunately, only two cases had radiological investigations done before appearing for pathological tests, and patients being lost to follow-up did not allow for access to the other patients' radiological data. 

FNAC yielded mucoid material in all cases. Cytological smears were evaluated for mucin content, cell arrangement, nuclear atypia, and vascular patterns (Table 2).

**Table 2:** Cytological features The numerical identifiers (e.g., 1, 2, 3, etc.) used in the table are arbitrary and were created solely for the purpose of referencing specific cases within this article. These identifiers do not correspond to any patient-identifying information.

Case number	Extracellular mucin	Cellularity	Cell arrangement	Atypia	Branching capillaries
Case 1	Abundant and intracytoplasmic	Abundant	Predominantly clustered	Mild to moderate	+
Case 2	Abundant and intracytoplasmic	Abundant	Larger: predominantly clustered; Smaller: both clustered and dispersed	Larger: mild; Smaller: moderate	+
Case 3	Abundant	Abundant	Predominantly clustered	Mild	-
Case 4	Abundant	Abundant	Predominantly clustered	Mild	+
Case 5	Abundant	Abundant	Predominantly clustered	Mild	+
Case 6	Abundant	Abundant	Predominantly clustered	Mild	+
Case 7	Abundant	Abundant	Predominantly clustered	Mild	+

**Figure 1:**
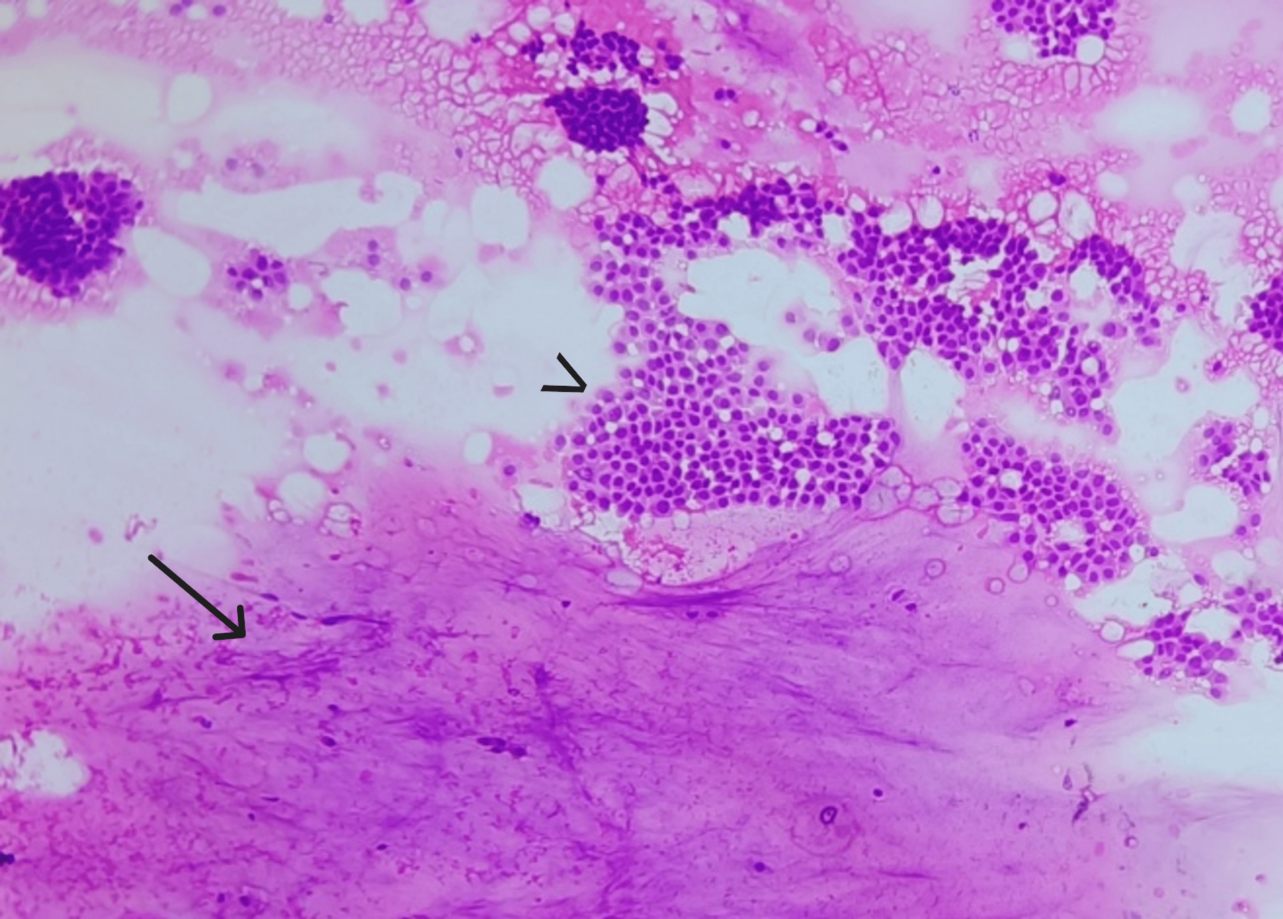
Smear shows abundant extracellular mucin (arrow) and cohesive clusters and sheets of epithelial cells (arrowhead) (H&E, 10X).

**Figure 2:**
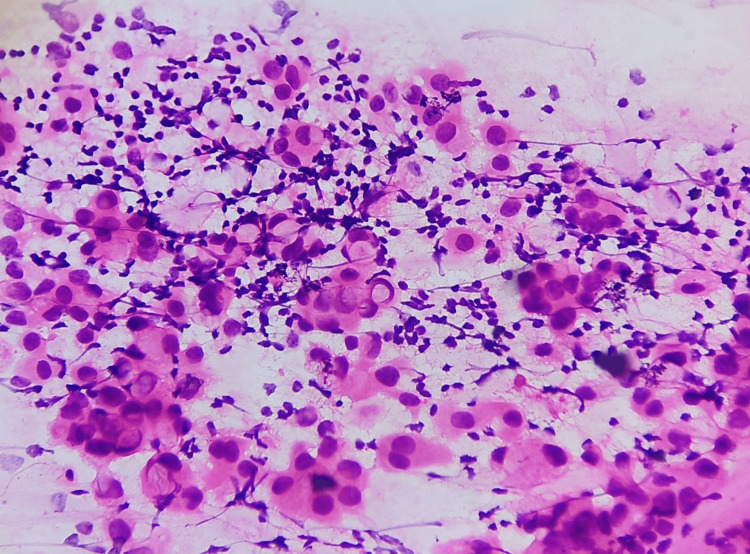
Smear shows cells with intracytoplasmic mucin compressing nuclei to the periphery (H&E, 40X).

**Figure 3:**
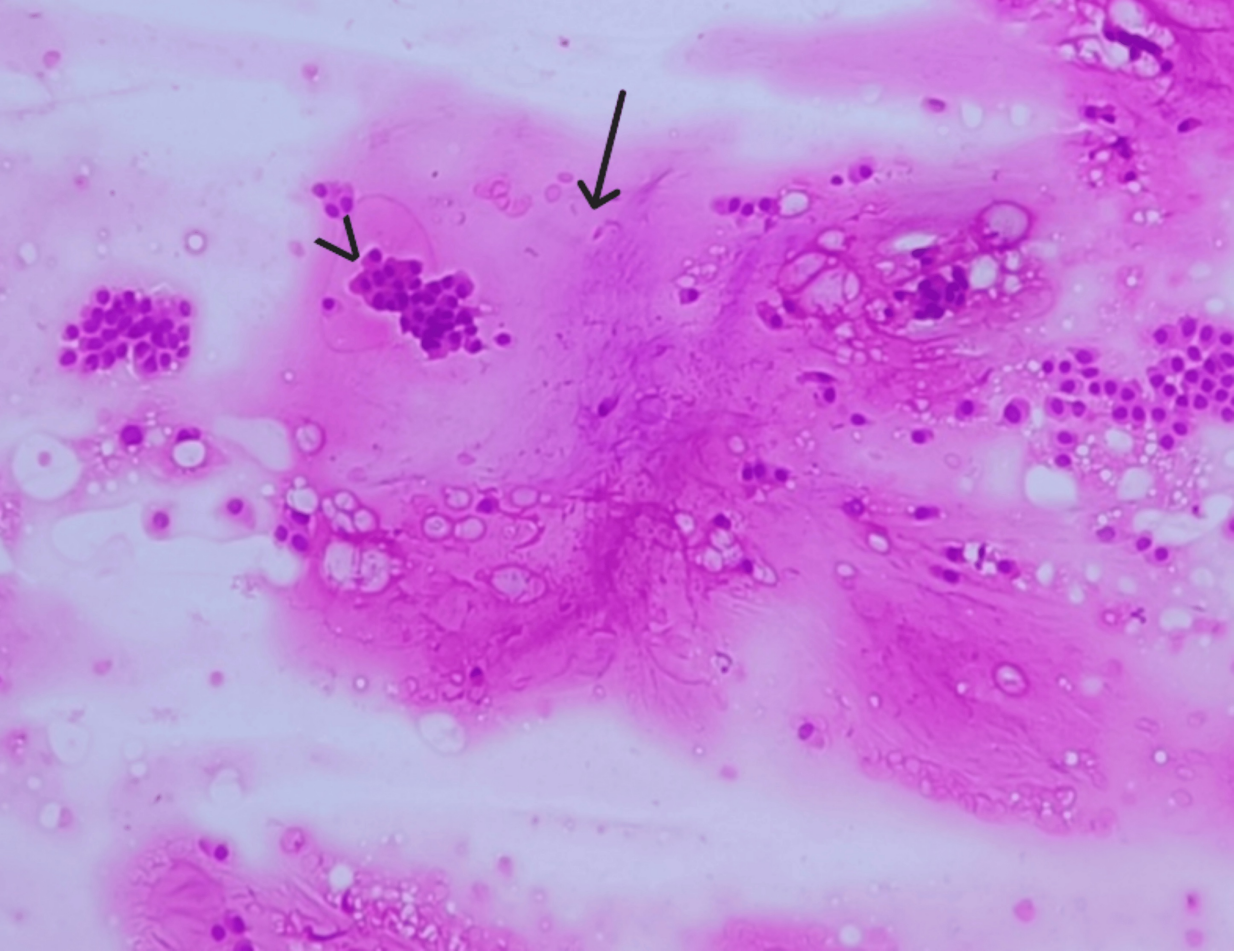
Smear shows pools of mucin (arrow) with floating islands of cells (arrowhead) (H&E, 10X).

**Figure 4:**
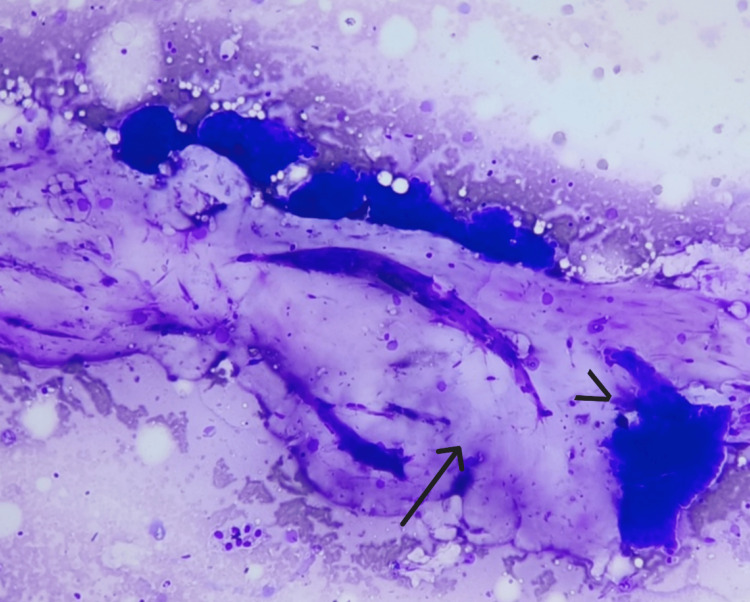
Smear shows pools of mucin (arrow) with tight clusters of cells (arrowhead) (MGG, 10X). MGG: May-Grunwald Giemsa

The smears revealed the following features: Abundant extracellular mucin was observed in all cases (Figures 1-4). The typical description of “pools of mucin with floating islands of cells” was noted. Two cases showed both extracellular (H&E stain (bluish pink), Papanicolaou stain (greenish), and MGG stain (pale blue)) and intracytoplasmic mucin.

**Figure 5:**
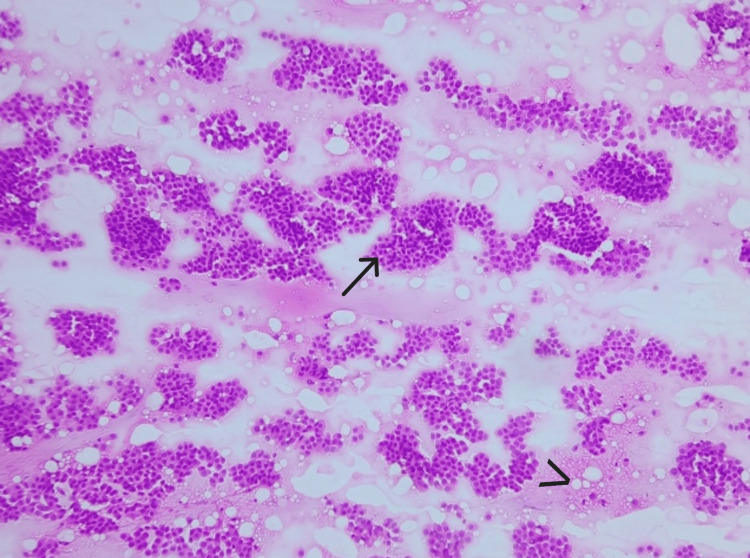
Smear shows abundant cellularity (arrow) and extracellular mucin (arrowhead) (H&E, 10X).

**Figure 6:**
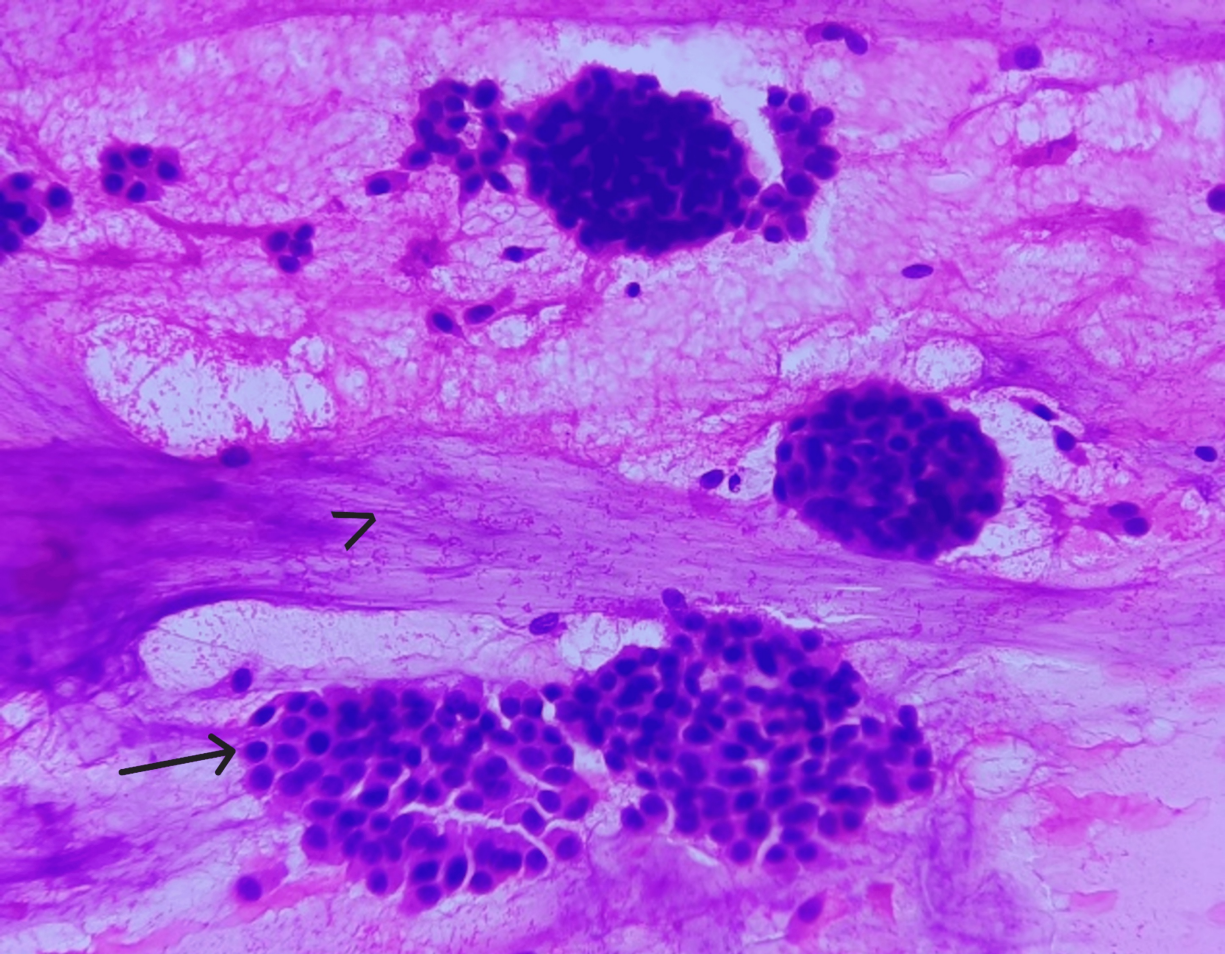
Smear shows tight clusters of epithelial cells (arrow) and extracellular mucin (arrowhead) (H&E, 40X).

Cellularity and cell arrangement are shown in Figures 5, 6. In all cases, there was abundant cellularity. Most of the epithelial cells were seen to be arranged predominantly in clusters. There were a few isolated cells dispersed in the mucin in all the cases.

**Figure 7:**
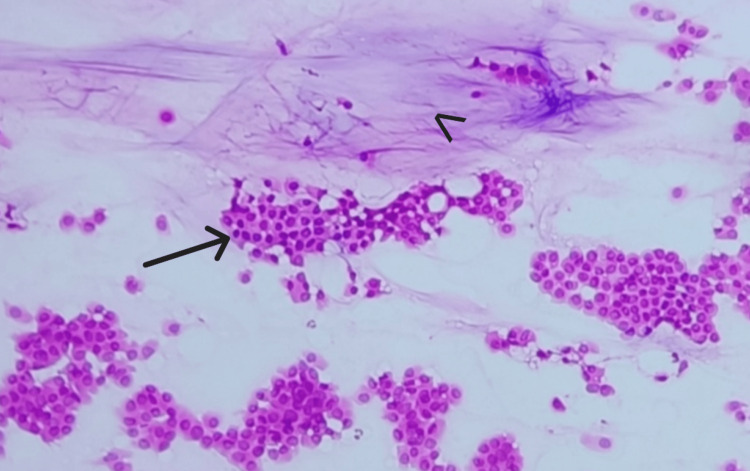
Smear shows sheets of uniform cells (arrow) and extracellular mucin (arrowhead) (H&E, 10X).

**Figure 8:**
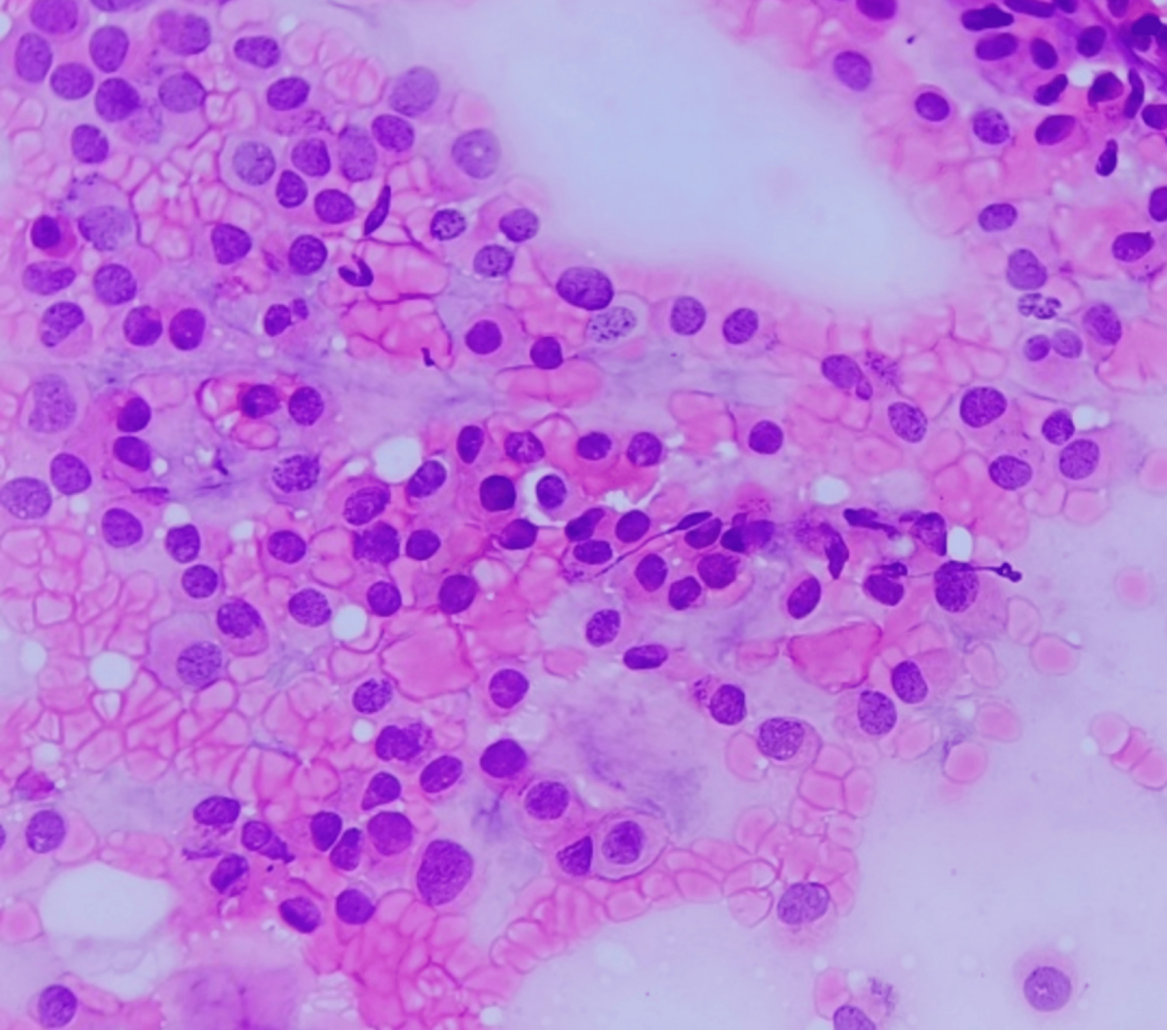
Smear shows dispersed epithelial cells with a moderate amount of cytoplasm and a round nucleus showing regular nuclear margins, fine granular chromatin, and indistinct nucleoli with minimal atypia (H&E, 40X).

Cell morphology is displayed in Figures 7, 8. In all cases, cells were uniform and had a moderate amount of cytoplasm and a round nucleus showing mild atypia. In two cases, intracytoplasmic mucin compressing the nuclei to the periphery was seen. Nuclei were predominantly round with regular nuclear margins, fine granular chromatin, and indistinct nucleoli. Mild to moderate atypia was noted in all cases (Figure 8).

**Figure 9:**
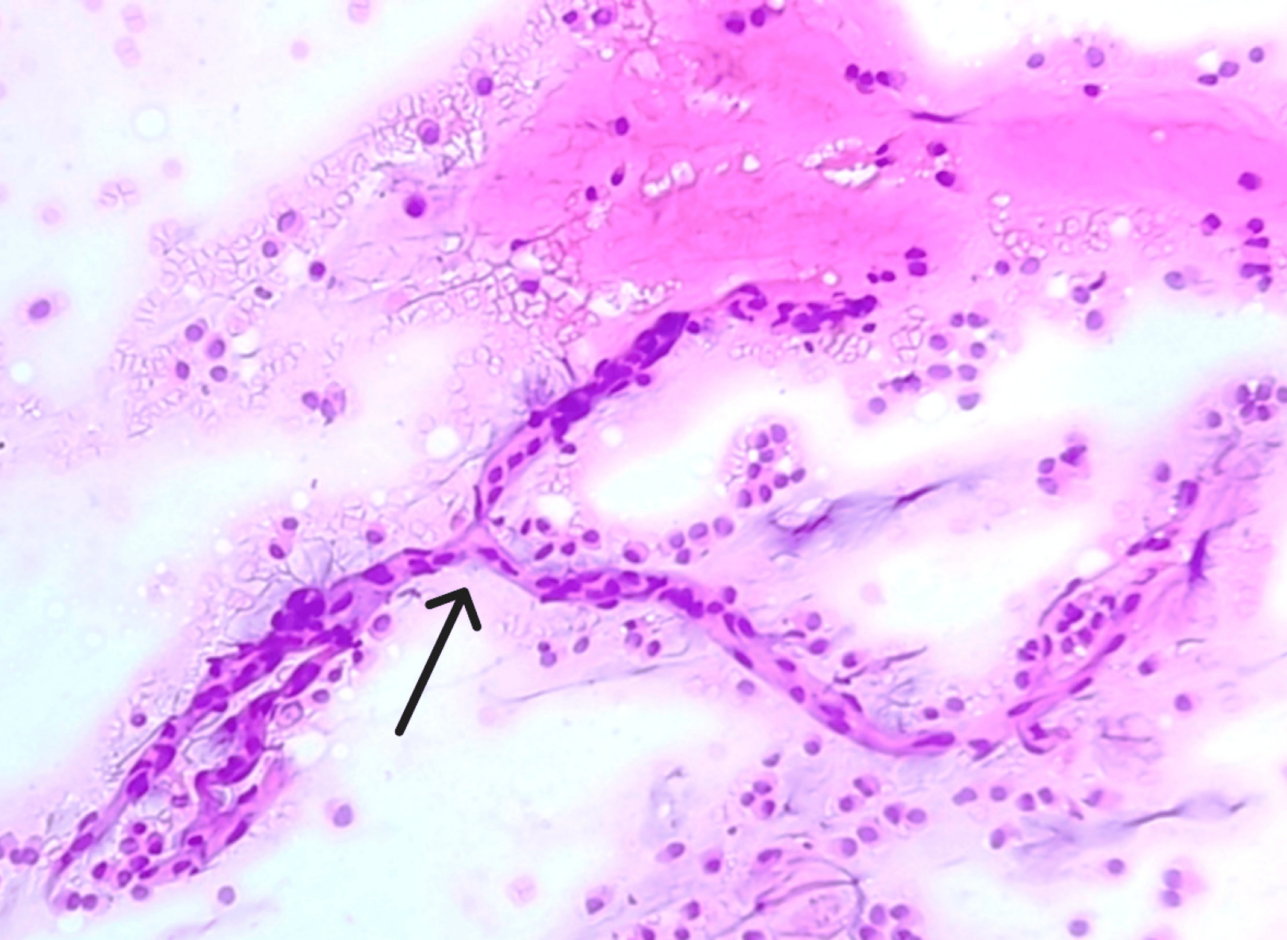
Smear shows branching delicate capillary fragment (arrow), surrounding mucin and few dispersed epithelial cells (H&E, 10X).

**Figure 10:**
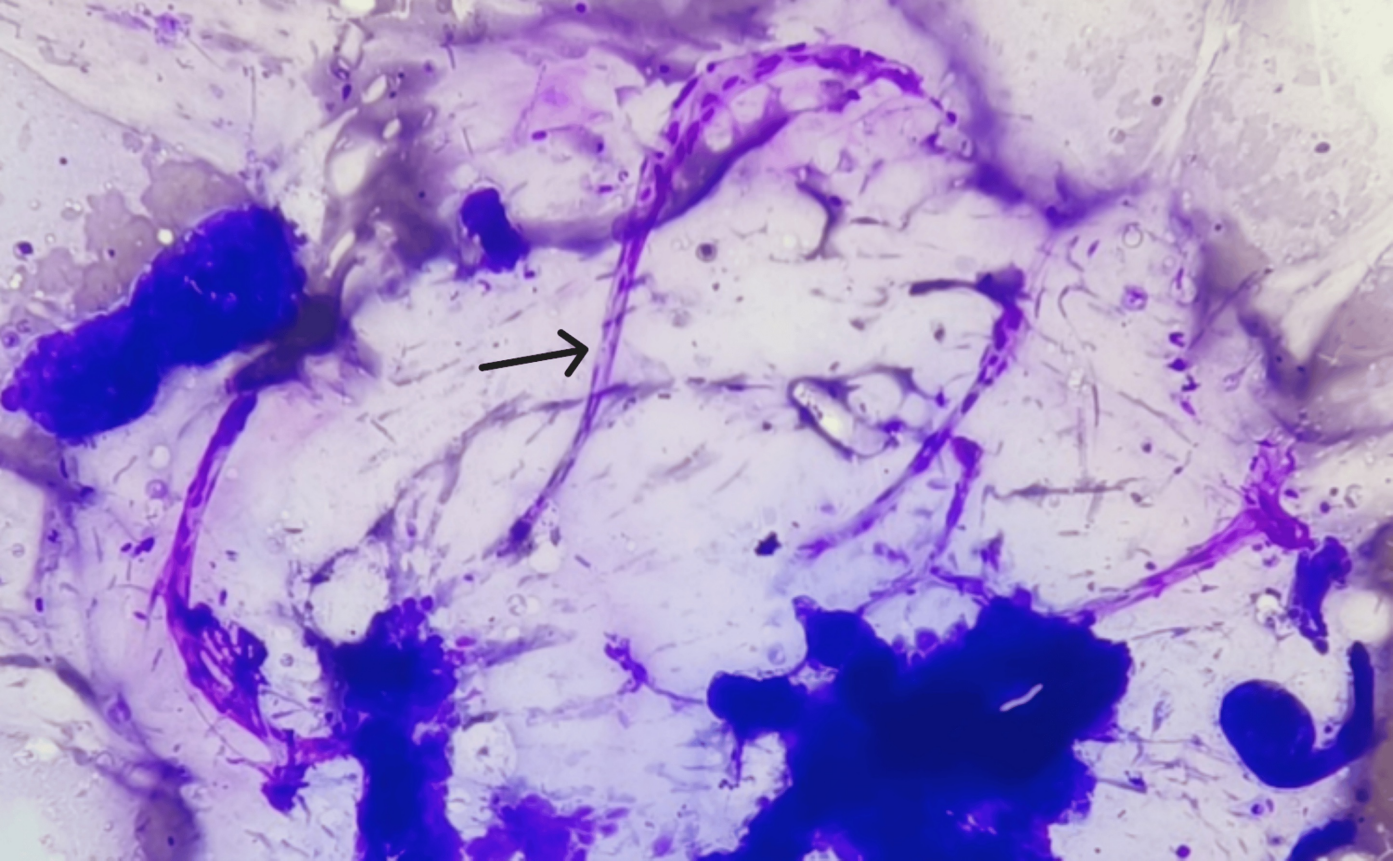
Smear shows branching delicate capillary fragments (arrow), surrounding mucin and tight clusters of epithelial cells (MGG, 10X). MGG: May-Grunwald Giemsa

Branching delicate capillary fragments (chicken-wire vascularity) were noted in six (85%) cases (Figures 9, 10).

**Figure 11:**
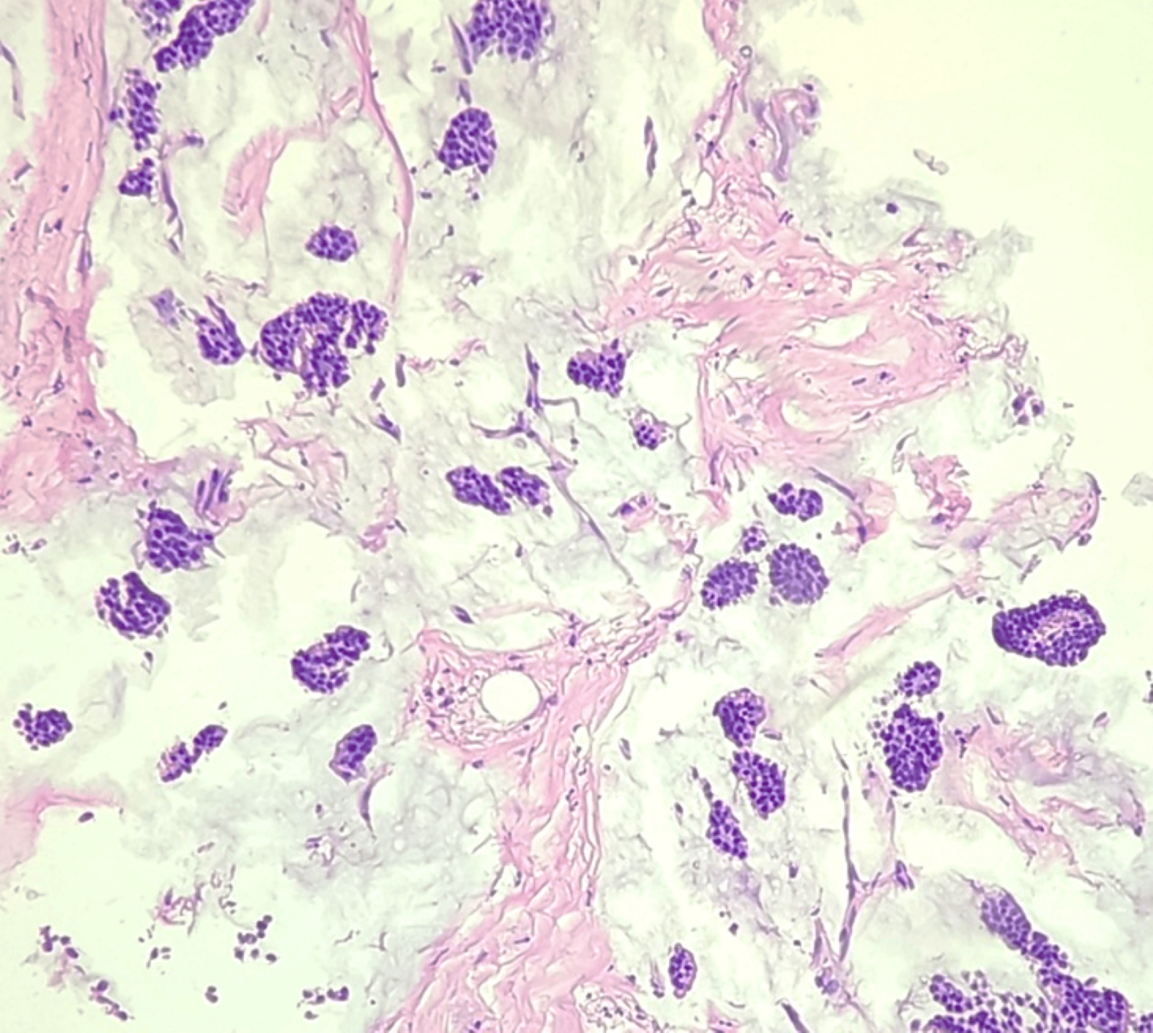
Section shows abundant mucin separated by fibrocollagenous stromal bands and clusters of epithelial cells (H&E, 10X).

**Figure 12:**
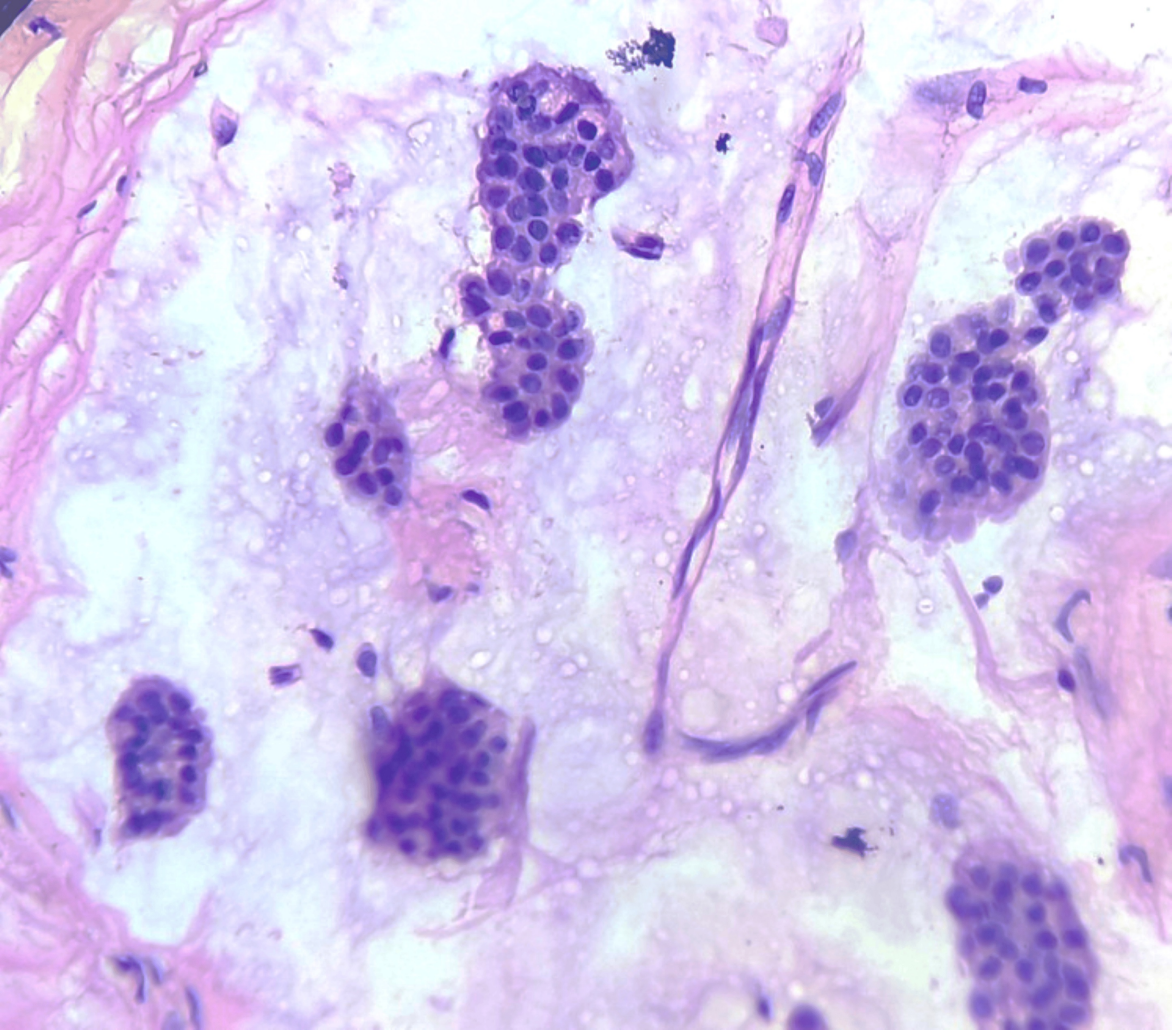
Section shows abundant mucin, branching capillary and tight clusters of epithelial cells showing minimal atypia (H&E, 40X).

Histopathological correlation was available for all cases, confirming pure mucinous carcinoma (Figures 11, 12). No false positives or negatives were noted on cytology.

There was a single case of metachronous bilateral breast mucinous carcinomas, with the right and left breast lesions being palpable five and three years back, respectively, by the patient. Both lesions were diagnosed cytologically and confirmed on histopathology. Both axillae remained free and devoid of any lymphatic metastases. There was another case in which two lumps were detected on ultrasound, though clinically, only one lump was noticed.

## Discussion

All cases in the present study were in the peri- or post-menopausal age group, which was consistent with previous studies [4-6]. The mean size of these lesions was also found to be congruent with previous research [4, 6]. Multiple studies have reported that the majority of cases of mucinous carcinoma of the breast exhibited abundant extracellular mucin [4-7]. All studies [4-7] have reported that the nuclear atypia in cases of mucinous carcinoma was mild, which is an essential WHO criterion [3]. The typical chicken-wire capillary branching was prominent in most of the present cases, which is a significant but not diagnostic clue towards the diagnosis of mucinous carcinoma, as noted in previous studies [4, 5].

In one case, the typical ultrasonographic finding of microlobulation was also noted, which has been found to be relatively specific for mucinous breast carcinomas [5].

Mucinous carcinomas are classified by WHO based on the percentage of tumor area involved by the typical mucinous morphology as pure mucinous carcinomas (>90%) and mixed mucinous carcinomas (10-90%). Typically, pure mucinous subtypes show abundant extracellular mucin with floating islands and isolated tumor cells within. Mixed mucinous carcinomas often show more solid areas with an invasive ductal carcinoma component [3]. Hence, it is imperative that FNAC be done from separate multiple areas in larger tumors to rule out any foci of duct carcinoma not otherwise specified, as was done in the present study. While it should be stressed that FNAC can never rule out the diagnosis of a mixed mucinous carcinoma, the presence of the same can be indicated by multiple aspirations from different locations within the same tumor. 

There are several case series and case reports of mucinous carcinoma of the breast. However, an extensive literature search yielded only two case reports of bilateral mucinous carcinoma of the breast, both of which were diagnosed on histopathology [8, 9]. In the present study, there was a single case of bilateral mucinous carcinoma of the breast, making it only the third case to be reported so far. Since this presentation is quite rare, care was taken to ensure clinical, radiological, and histopathological signs were conforming with the diagnosis of mucinous carcinoma. It is the first case of mucinous carcinoma presenting in bilateral breasts to be reported on FNAC. Bilateral breast carcinomas have been previously associated with the mucinous variant; however, the contralateral lesion has either been lobular carcinoma or lymphoma [10, 11].

As per a study conducted in 2019, Begg et al. found that around 6% of cases of bilateral breast carcinomas are metastases in one breast from the contralateral breast [12, 13]. However, considering the rarity of mucinous carcinoma, there is no definitive research or information regarding metastasis to the contralateral breast in mucinous breast carcinomas. Moreover, mucinous carcinomas are known to have a lower risk of metastasis [3]. Hence, it is unlikely that the present case of bilateral mucinous carcinomas is the result of metastasis from the contralateral breast. Hence, this case was considered a metachronous bilateral mucinous carcinoma of the breast, which has not been reported on cytology in the literature.

There are several close differential diagnoses that need to be considered and ruled out prior to reporting a case as mucinous carcinoma on cytology.

Invasive ductal carcinoma with mucinous component is the closest differential to pure mucinous carcinoma. But sampling from multiple sites is imperative for proper representation. Additionally, the presence of loss of cohesiveness resulting in a dispersed population of tumor cells and higher degrees of nuclear atypia are indicators of mixed mucinous carcinoma [3]. Hence, when these features are seen, a cytology report suggesting duct carcinoma with a mucinous component and confirmation by histopathology is preferable.

Mucocele-like lesions usually yield scant cellularity, minimal atypia, and attached myoepithelial cells to epithelial clusters. Carcinomas with signet ring cell differentiation only show intracytoplasmic mucin with signet ring morphology, with absent extracellular mucin. Fibroadenoma and phyllodes tumor with myxoid change have been described as close differentials. But they can be cytologically distinguished by the presence of bare-bipolar nuclei in the background, tight antler-patterned cohesive epithelial clusters, and the magenta-colored myxoid material best appreciated with MGG stain [6].

## Conclusions

Strict adherence to the above diagnostic cytologic criteria, which includes abundant pools of mucin, tight clusters of epithelial cells exhibiting mild atypia, and branching capillaries, is the key to a confident diagnosis of mucinous carcinoma of the breast on cytology. Additionally, correlation with the clinical presentation of a slow-growing tumor and FNA from multiple sites in larger tumors helps avoid a cytological misdiagnosis. Though extremely rare, bilateral mucinous carcinoma of the breast does occur and can be reliably diagnosed on cytology.
